# Time computations in anuran auditory systems

**DOI:** 10.3389/fphys.2014.00206

**Published:** 2014-05-30

**Authors:** Gary J. Rose

**Affiliations:** Department of Biology, University of UtahSalt Lake City, UT, USA

**Keywords:** temporal processing, whole-cell patch, inferior colliculus, midbrain, acoustic communication, time coding, iontophoresis, transformations

## Abstract

Temporal computations are important in the acoustic communication of anurans. In many cases, calls between closely related species are nearly identical spectrally but differ markedly in temporal structure. Depending on the species, calls can differ in pulse duration, shape and/or rate (i.e., amplitude modulation), direction and rate of frequency modulation, and overall call duration. Also, behavioral studies have shown that anurans are able to discriminate between calls that differ in temporal structure. In the peripheral auditory system, temporal information is coded primarily in the spatiotemporal patterns of activity of auditory-nerve fibers. However, major transformations in the representation of temporal information occur in the central auditory system. In this review I summarize recent advances in understanding how temporal information is represented in the anuran midbrain, with particular emphasis on mechanisms that underlie selectivity for pulse duration and pulse rate (i.e., intervals between onsets of successive pulses). Two types of neurons have been identified that show selectivity for pulse rate: long-interval cells respond well to slow pulse rates but fail to spike or respond phasically to fast pulse rates; conversely, interval-counting neurons respond to intermediate or fast pulse rates, but only after a threshold number of pulses, presented at optimal intervals, have occurred. Duration-selectivity is manifest as short-pass, band-pass or long-pass tuning. Whole-cell patch recordings, *in vivo*, suggest that excitation and inhibition are integrated in diverse ways to generate temporal selectivity. In many cases, activity-related enhancement or depression of excitatory or inhibitory processes appear to contribute to selective responses.

## Introduction

Anuran amphibians have long served as models for investigating how acoustic communication signals are represented and processed in the auditory system. Much recent attention has been focused on how time computations are performed. Temporal complexity in anuran vocalizations varies considerably across calls of different species and within the calls of individual species. In the simplest calls, signal frequency and amplitude remain relatively constant, and the temporal structure is defined mainly by its duration. Fundamentally, the temporal structure of sound consists of how the frequency and amplitude of a signal vary over time. Temporal features of vocalizations include call duration, intracall amplitude and or frequency modulation, and combinations of structurally differentiated, phrase-like elements. Most commonly, calls consist of a series of pulses that have a specific shape and duration. In many cases, the calls of closely related species are spectrally highly similar, but differ markedly in their temporal structure. Thus, recognition of temporal features is essential for differentiating between intraspecific and heterospecific calls.

Behavioral studies have shown that anurans are able to discriminate between calls that differ almost exclusively in temporal structure (Gerhardt, 2001). Discrimination can be based on pulse rate (PR) or rate of amplitude modulation (AM), pulse duration, pulse shape, direction and rate of frequency modulation, and number of acoustic elements. Also, longer calls tend to be favored, even when alternatives that are atypically short in duration are repeated more often to maintain equal total stimulus energy (Klump and Gerhardt, 1987); this preference appears to saturate in the range of durations seen for calls of males in the breeding season (Ward et al., 2013). Remarkably, inserting a gap in a sequence of regularly repeated pulses can markedly reduce call attractiveness (Schwartz et al., 2010). These discriminative capacities are important in the reproductive biology of anurans, and are under the forces of natural and sexual selection. Correspondingly, anurans represent a fascinating and superb system in which to investigate neural specializations for performing temporal computations.

Peripherally, the temporal structure of sounds is represented in the spatio-temporal patterns of spike activity of auditory-nerve fibers. For example, in the case of AM signals, the periodic fluctuations of sound amplitude (sound pulses) are represented/coded in the periodicity of spikes in auditory-nerve fibers (Rose and Capranica, 1985). At the level of the midbrain (anuran inferior colliculus, IC), however, evidence of a major transformation exists such that many neurons show AM (or pulse rate) tuning (i.e., cells respond best over a narrow range of AM or pulse rates) (Rose and Capranica, 1983). Other cells have been recorded that show selectivity for sound duration (e.g., short-pass neurons respond to tone bursts only if sound duration does not exceed a particular value); other cells show band-pass or long-pass characteristics. The primary purpose of this review is to summarize recent progress toward identifying the types of transformations in temporal coding that exist and elucidating the mechanisms that underlie neural selectivity for temporal features of sound.

## Representations and computations relating to AM or pulse rate, and duration

### Selectivity for AM or pulse rate

Using sinusoidal AM stimuli, temporally selective neurons in the anuran IC show either low-pass, high-pass, band-pass or band-suppression characteristics (Rose and Capranica, 1983; review: Rose and Gooler, 2007). In this stimulus, each modulation cycle can be considered to be a sound pulse; as AM rate increases, holding stimulus duration constant, pulse duration and rise/fall times decrease, and the number of pulses increases. Selectivity for AM rate, therefore, can be a consequence of sensitivities to any one or a combination of these temporal attributes. For example, cells that respond phasically to sound pulses show high-pass or band-pass selectivity for AM rate; below the best AM rate, the response level decreases with AM rate because pulse number also decreases. Many band-pass neurons, however, fail to respond to slow AM rates, suggesting that this AM selectivity class is mechanistically heterogeneous. A clearer picture of the temporal selectivities of cells emerges from also evaluating responses to stimuli in which pulse rate is varied while holding pulse duration, shape and number constant. Neurons that show selectivity for pulse rate largely comprise two groups. “Long-interval” cells respond well to slow PRs, but weakly, if at all, to fast rates (Alder and Rose, 2000). Neurons of this type appear to be particularly common in the midbrain auditory region (laminar N.) of the aquatic frog *Xenopus laevis* (Elliott et al., 2011). Other neurons respond best to mid or fast PRs, in a strongly band-pass fashion in some cases, and are known as “interval-counting neurons” (Alder and Rose, 1998; Edwards et al., 2002). Interval-counting neurons respond after a particular number of sound pulses have occurred with optimal timing; stimuli are ineffective if the interval between onsets of successive pulses alternates between values that are substantially shorter or longer that the optimal interval (Edwards et al., 2002, 2007). Thus the responses of these cells reflect the number of consecutive correct intervals, not the number of pulses that have occurred in a particular time window. Remarkably, a single interval that is too long can reset the counting process. As mentioned earlier, some interval-counting neurons show band-suppression characteristics when tested with sinusoidal AM stimuli. These cells are a subset of the interval-counting population and respond to slow AM rates, or tone bursts of sufficient duration, apparently because these stimuli elicit appropriately timed patterns of presynaptic spikes (Edwards and Rose, 2003; Leary et al., 2008). Band-suppression neurons also have been recorded in the IC of mice (Geis and Borst, 2009), but it is unclear whether they show interval-counting properties.

### Mechanisms of interval selectivity

Advances in performing whole-cell patch recording *in vivo* (Ferster and Jagadeesh, 1992; Rose and Fortune, 1996) paved the way for investigating processes that mediate transformations in the representations of temporal information (i.e., the mechanisms that underlie temporal computations). More generally, this methodology has led to a renaissance in elucidating the integrative mechanisms that underlie response selectivity in central nervous systems. To make these recordings, a patch-type pipette is used to make a gigaohm seal onto a cell; the membrane that is invaginated into the pipette tip is then ruptured to enable recording of the transmembrane potential of the cell.

**Figure 1:**
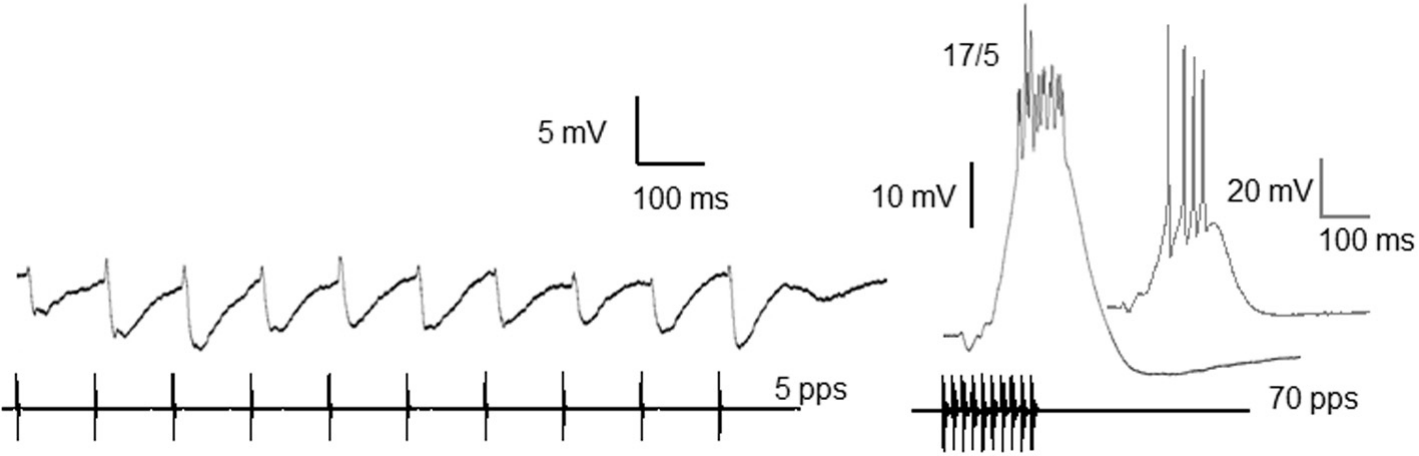
**Whole-cell recordings of responses from an interval-counting neuron to stimulus pulses presented at 5 and 70 pulses/s**. Averaged traces (black) and a single unaveraged trace (gray, inset) are shown. Ratios: number of spikes over the number of stimulus presentations. Resting potential = −68 mV; carrier frequency = 300 Hz; 66 dB SPL. Adapted from (Rose et al., 2011).

**Figure 2:**
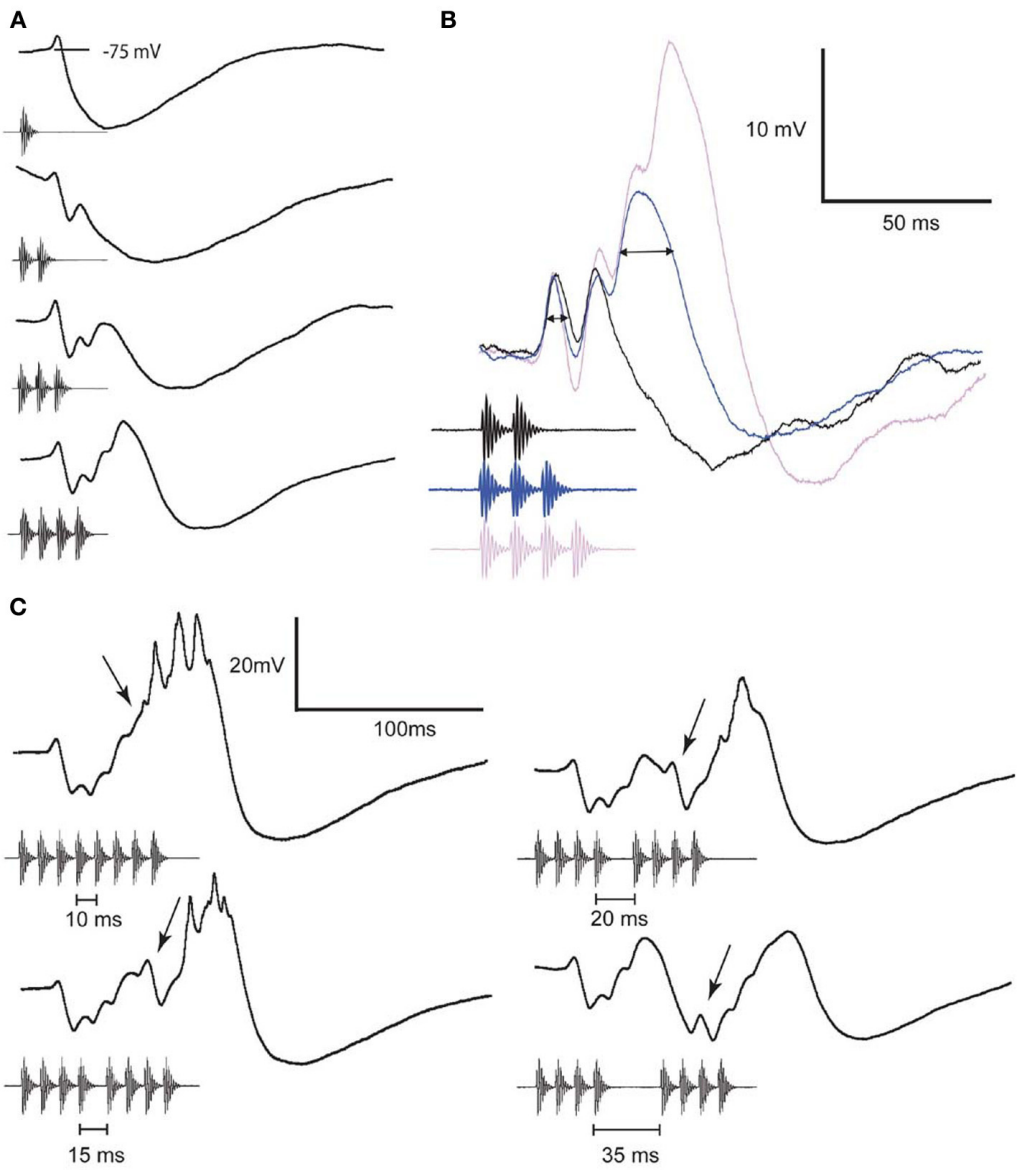
**Averaged whole-cell recordings from an interval-counting neuron to stimuli that differed in pulse number (A,B) or the duration of a middle interval in a pulse sequence (C)**. Responses shown in **(B)** are from recordings made while hyperpolarizing the neuron by approximately 12 mV (negative current-clamp mode). Horizontal arrow denotes EPSP duration at half-maximal amplitude; enhancement of EPSP amplitude is first observed to the 3rd pulse. Arrows in **(C)** indicate the EPSP elicited by the 5th pulse in each stimulus. The interval-counting process was reset by an interval of 35 ms, hence no spikes were elicited by the stimulus. Spikes were elicited by the other stimuli and appear as small peaks because of averaging. Resting potential = −70 mV, threshold = 49 dB SPL, and BEF = 800 Hz. Adapted from (Edwards et al., 2007).

Whole-cell patch recordings from interval-counting neurons have revealed a complex interplay of inhibition and excitation (Edwards et al., 2007). In the most selective cases, individual sound pulses, presented at slow rates, elicit primarily IPSPs (Figures 1, 2A). As additional pulses are presented at the optimal rate, however, excitation progressively overcomes inhibition (Figures 2A–C) and spikes are elicited if a sufficient number of pulses (5 in the case shown) are presented. Thus, stimuli in which successive pulses have intervals that are alternately shorter or longer than optimal are ineffective, and elicit primarily inhibition. Remarkably, and consistent with the behavioral results mentioned earlier (Schwartz et al., 2010), a single long interval (2–3 times optimal) that is embedded in a series of optimally timed pulses can completely reset the interval-counting process (Figure 2C). The rate-dependent enhancement of excitation, which plays a critical role in the interval selectivity and counting properties of these neurons, appears to be reset by a long interval (arrows, Figure 2C); the first pulse following the gap elicits a small EPSP and large IPSP, shifting the balance once again in favor of the inhibition. The activity-dependent enhancement of excitation in interval-counting neurons stands in marked contrast to that seen in long-interval cells, which we turn to next.

**Figure 3:**
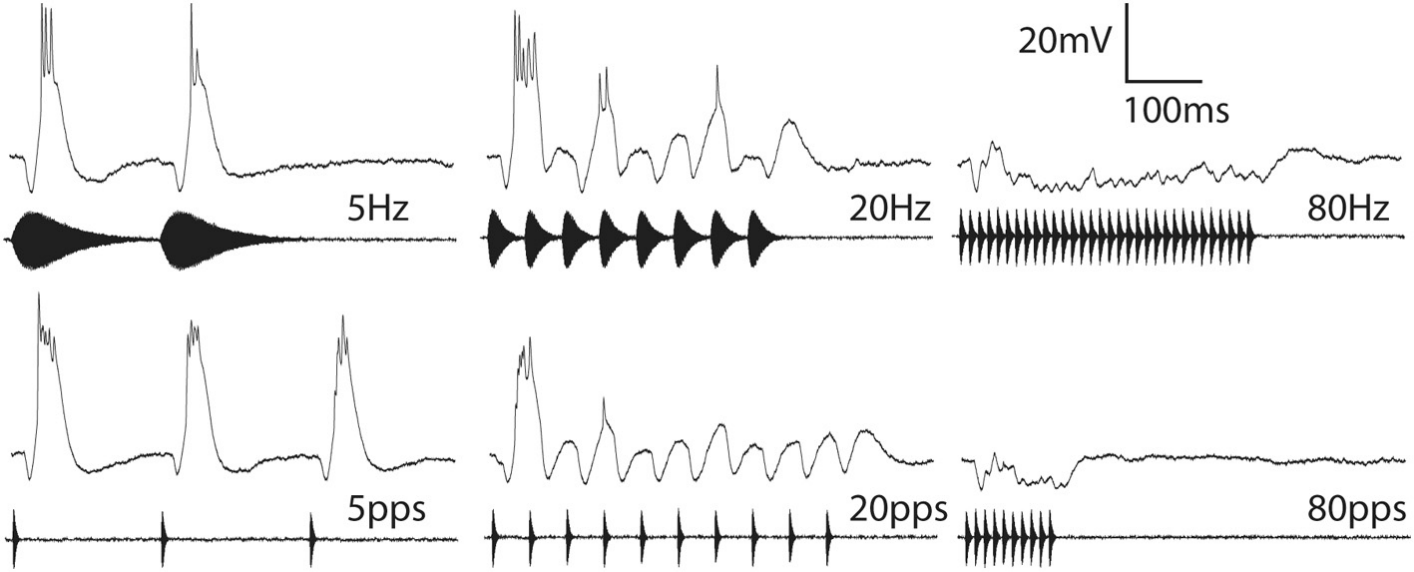
**Averaged responses from a long-interval neuron to stimuli in which pulse duration varied (top) or was held constant at 10 ms (bottom) across AM rates (Hz) or pulse rates (pulses/s) of 5, 20 or 80**. Resting potential = −69 mV; BEF = 1300 Hz; 53 dB SPL. Adapted from (Edwards et al., 2008).

Long-interval neurons respond well to pulses that are presented individually or at slow rates. Fast pulse rate stimuli, however, elicit a phasic onset response, or, in the most selective cases, no spikes at all (Alder and Rose, 2000; Edwards et al., 2008). Whole-cell recordings from long-interval cells have revealed that individual pulses trigger suprathreshold depolarizations (EPSPs) and phasic inhibition (Figure 3), consistent with a model proposed by Grothe (1994). In cells that do not respond to fast PRs, inhibition appears to precede excitation. At fast PRs, the inhibition triggered by a pulse appears to overlap temporally with the longer-latency excitation from the preceding pulse, thereby preventing excitation from triggering spikes. For neurons that show phasic responses to the onset of fast PR stimuli, inhibition appears to follow excitation. In addition, rate-dependent depression of excitation may contribute to the phasic, onset response properties of these cells to fast PR stimuli.

### Duration selectivity

**Figure 4:**
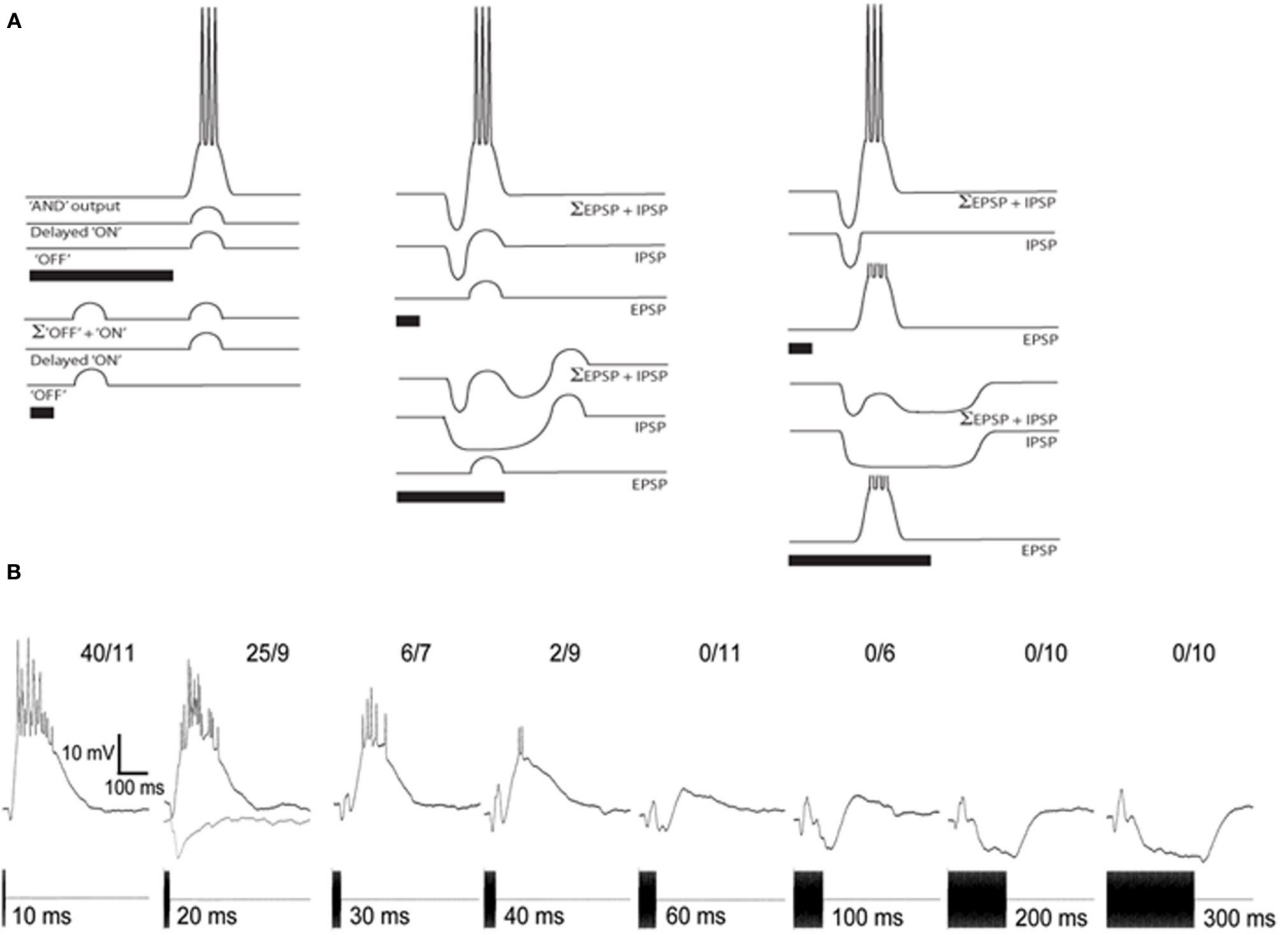
**(A)** Models of duration selectivity proposed by (Narins and Capranica, 1980) (left); (Casseday et al., 1994) (middle); and (Fuzessery and Hall, 1999) (right). For each stimulus condition, traces depict the excitation alone (EPSP), inhibition alone (IPSP), and combined (Σ EPSP + IPSP). **(B)** Averaged responses of a short-pass neuron to tone-bursts ranging in duration from 10 to 300 ms. Inhibition was isolated by examining the responses to 20 ms, 300 Hz tone bursts (gray, lower trace), a stimulus that evoked minimal excitation. Resting potential = −87 mV; BEF = 750 Hz; 65 dB SPL. Adapted from (Leary et al., 2008).

Peripherally, the duration of a tone burst is coded in the duration of activity of auditory-nerve fibers, with some units showing adaptation of firing rates during the stimulus (Megela and Capranica, 1981). However, in the anuran IC there is evidence of a transformation in this representation, such that neurons show short-pass, band-pass or long-pass duration selectivity (Narins and Capranica, 1980; Gooler and Feng, 1992). Similarly, duration selectivity is also present in the IC of bats (Casseday et al., 1994; Fuzessery and Hall, 1999; Macias et al., 2011), and mice (Brand et al., 2000), and appears to be a general feature of vertebrate auditory systems (Aubie et al., 2012). Several models have been proposed for how duration selectivity might be generated (Figure 4A). Stimulus onset might elicit delayed excitation that summates with “off excitation” (Figure 4A, left) over a particular range of stimulus durations. Alternatively, duration selectivity might result from interplay between short-latency, tonic inhibition and delayed, phasic excitation that is either subthreshold or suprathreshold (Figure 4A middle and right panels, respectively); in the first case, post-inhibitory rebound summates with the delayed, subthreshold excitation to trigger spikes. For short-pass or band-pass cells in anurans and bats, responses occur after the end of the stimulus; thus response latency increases with tone burst duration (Faure et al., 2003; Leary et al., 2008). In bats, blockade of inhibition decreases response latency and broadens the range of durations over which the neuron responds, suggesting that short-latency, tonic inhibition plays an important role in generating short-pass and band-pass duration selectivity (Casseday et al., 1994).

Whole-cell recordings from short-pass duration-selective neurons in the anuran IC (Leary et al., 2008) support the hypothesis that tone bursts elicit short-latency inhibition, followed by delayed excitation (Figure 4B). For the cell shown, each stimulus elicited an initial hyperpolarization, followed by a depolarization that, for tone-burst durations of 10–30 ms, was sufficient for reliably eliciting spikes. The short-latency nature of the inhibition was particularly evident in the responses to 300 Hz tone bursts, 20 ms in duration, which elicited little excitation (gray, lower trace, Figure 4B), The duration of hyperpolarization in most cases increases with tone burst duration, suggesting that short-pass neurons receive tonic inhibition. The delayed excitation, however, appears to be phasic (i.e., relatively invariant in time course and magnitude across a wide range of tone burst durations). It is presently unclear whether rebound from inhibition might sum with delayed excitation to augment responses for short- or mid-duration tone bursts. Band-pass duration selectivity also appears to result from interplay between short-latency, tonic inhibition and delayed excitation, however the strength of the excitation appears to increase non-linearly with tone burst duration until the best duration is reached.

Two types of long-pass duration selectivity have been identified (Leary et al., 2008). One class of long-pass duration-selective neurons also responds well to a series of sound pulses, and show interval-counting properties. These neurons show band-suppression characteristics for sinusoidal AM stimuli. It appears that long-duration tone bursts elicit a temporal pattern of spikes in the presynaptic excitatory inputs that closely match that of a series of pulses. The other class of duration long-pass cells responds to long-duration tone bursts, but not to pulses presented at fast rates (i.e., these neurons do not show interval-counting properties). The mechanism underlying this type of long-pass duration selectivity is not well understood, however our working hypothesis is that it may result from the integration of phasic inhibition and tonic excitation. In this model, short-duration tone bursts elicit primarily inhibition. As tone burst duration increases, however, inhibition wanes and tonic excitation promotes depolarization, which, over time, is sufficient for triggering spikes.

## Conclusions and future directions

Transformations in representations of the temporal structure of acoustic signals appear to be fundamental to auditory function. These transformations are manifest in the anuran IC as a population of neurons that respond selectively for particular temporal features of sounds. Selectivity for tone burst duration, and pulse or AM rate has been observed. Similarly, selectivity for these temporal features has been shown in the IC of mammals (reviewed in Casseday et al., 2002; Macias, Faure this volume).

Although the mechanisms that underlie these transformations have been difficult to uncover, recent whole-cell, and conventional intracellular, recordings from IC neurons *in vivo* have provided new windows into the integrative processes that contribute to temporal selectivity. These recordings, in anurans and mammals (Casseday et al., 1994; Geis and Borst, 2009; Gittelman et al., 2009; Geis and Borst, 2013), have begun to shed light on how activity-dependent excitation and inhibition can be integrated in diverse ways to generate selectivity for temporal features of biologically relevant sounds. For interval-counting neurons, rate-dependent enhancement of excitation appears to overcome inhibition in the optimal range of pulse rates, in general agreement with a model of interval selectivity proposed by Buonomano (2000); it is presently unclear whether rate-dependent depression of inhibition also contributes to interval counting and selectivity in IC neurons. Interplay between inhibition and excitation also appears to underlie long-interval selectivity; in this case, however, excitation appears to show rate-dependent depression. Rate-dependent depression of excitation may be a property that is communicated to the IC from the superior olive; approximately 27% neurons in this structure show low-pass or band-pass selectivity for AM stimuli (Condon et al., 1991). Similarly, inhibitory inputs may originate from other brainstem regions and/or local inhibitory interneurons. Interestingly, intrinsic biophysical properties of neurons (e.g., input resistance and time constant) (Yang et al., 2009) appear to not differ appreciably across neurons of different temporal selectivity classes (Tan and Borst, 2007), but may enhance selectivity as measured from spike output (Geis and Borst, 2009; Rose et al., 2011).

Future studies, employing additional experimental techniques, should provide further insights into the mechanisms that underlie temporal selectivity. Whole-cell recordings at several levels of current clamp (constant negative current injected to hyperpolarize the cell) can be used to mathematically extract the time courses of excitatory and inhibitory conductances (Priebe and Ferster, 2005), provided that voltage-dependent conductances are minimal. The time courses and magnitudes of conductances should provide a better understanding of how excitation and inhibition are integrated to generate responses selectively to particular temporal parameters of sounds. A particularly powerful and promising direction for future work is to perform whole-cell recording in conjunction with iontophoretic application of pharmacological agents (Rose et al., 2013). In this methodology, glutamate iontophoresis is used to position a multibarrel pipette close to the neuron from which whole-cell recordings are being made. Iontophoresis of specific receptor antagonists or agonists can be used to determine the roles that particular types of excitatory or inhibitory inputs play in generating temporal selectivity. Future studies could also employ newly developed optogenetic methods for activating particular groups of neurons in temporally defined patterns while recording from individual neurons.

Finally, comparative studies in anurans offer opportunities to investigate how additional time computations are performed and how neural circuits are modified during the course of evolution to generate novel temporal processing capabilities. For example, the two sister species of gray treefrogs *H. chrysoscelis* and *H. versicolor* produce calls that differ in the duration, shape and rate of repetition of sound pulses. *H. versicolor*, which evolved from *H. chrysoscelis* via polyploidy, shows the novel ability to discriminate between sounds that differ only in pulse shape; behavioral and neural preference for pulses that rise slowly in amplitude has been observed (Diekamp and Gerhardt, 1995). *H. chrysocelis* evaluates pulse rate *per se*, whereas the preference of *H. versicolor* for slower pulse rates appears to be based largely on their underlying preference for pulses of long duration and slow rise (Schul and Bush, 2002) Studies of gray treefrogs, including autopolyploids produced in the lab (Tucker and Gerhardt, 2012), offers the opportunity, therefore, to investigate the computations that underlie pulse-shape selectivity and how neural circuits change during the course of evolution.

### Conflict of interest statement

The author declares that the research was conducted in the absence of any commercial or financial relationships that could be construed as a potential conflict of interest.
